# New prospects on cerebellar reserve: Remarks on neuroprotective effects of experience in animals and humans

**DOI:** 10.3389/fnsys.2022.1088587

**Published:** 2023-01-06

**Authors:** Francesca Gelfo, Laura Serra, Laura Petrosini

**Affiliations:** ^1^Department of Human Sciences, Guglielmo Marconi University, Rome, Italy; ^2^IRCCS Fondazione Santa Lucia, Rome, Italy

**Keywords:** cerebellar reserve, brain/cognitive/neural reserve, cerebellum, cognition, neuroprotection, environmental enrichment, humans, animal models

## Abstract

The ability of the brain to change structure and function in response to experience accounts for its ability to successfully adapt to the environment in both learning processes and unique phases, such as during development and repair. On this basis, the occurrence of the brain, cognitive, and neural reserves has been advanced to explain the discrepancies between the extent of neurological damage and the severity of clinical manifestations described in patients with different life span experiences. Research on this topic highlighted the neuroprotective role of complex stimulations, allowing the brain to better cope with the damage. This framework was initially developed by observing patients with Alzheimer's disease, and it has since been progressively expanded to multifarious pathological states. The cerebellum is known to be particularly responsive to experience through extensive plastic rearrangements. The neuroprotective value exerted by reserve mechanisms appears to be suitable for basic neuronal plasticity in the cerebellum. Thus, it is of primary interest to deepen our understanding of how life experiences modify individuals' cerebellar morphology and functionality. The present study is aimed at analyzing the evidence provided on this topic by animal and human studies. For animals, we considered the studies in which subjects were submitted to enhanced stimulations before the damage occurred. For humans, we considered studies in which previous lifelong high-level experiences were associated with superior cerebellar abilities to cope with injury. Detailed indications of the processes underlying cerebellar reserves may be important in proposing effective interventions for patients suffering from pathologies that directly or indirectly damage cerebellar functionality.

## 1. Introduction

In neurological disorders, the underlying neuropathology and the clinical/cognitive/behavioral symptoms may not be directly related. Indeed, subjects may suffer from severe cerebral damage without corresponding severe functional impairment and vice versa. To explain this gap, Stern proposed the *reserve hypothesis* ~30 years ago (Stern et al., 1992). According to this hypothesis, a number of experiential factors can exert a protective action on the brain by making it more resilient to neurological damage. Such a protective action is possible since the brain is characterized by high-level plasticity that is present throughout the entire life span (Stern, 2009, 2012; Barulli and Stern, 2013).

Several activities are considered reserve builders. In particular, human studies have shown that various cognitive (reading, painting, writing, etc.), social (volunteering, participating in group leisure activities, marital status, etc.), and physical (running, walking, swimming, biking, etc.) activities; healthy lifestyle habits (diet, smoking, drinking, etc.); and education and occupational attainment, alone or in combination, build a reserve warehouse that can be spent to withstand physiological and/or pathological cognitive decline processes (Serra et al., 2018; Serra and Gelfo, 2019). These factors construct a sort of reserve in terms of structure (*brain reserve*: brain volume, parenchymal fraction, gray/white matter volume, lesion load, cortical thickness, gyrification, etc.) and function (*cognitive reserve*: memory, language, executive functions, learning, general cognition, etc.) of the brain, as well as the interaction between them (*neural reserve*: brain network efficiency, capacity, or flexibility; metabolism; etc.). The integrity of the structure leads to increased efficiency of the neural networks that in turn produce more efficient cognitive functions (Serra et al., 2017a, 2018).

In animal models, several studies have been performed on the neuroprotective effects of exposure to enhanced stimulations before the occurrence of brain damage by using the classical paradigm of environmental enrichment (Diamond et al., 1966; Rosenzweig et al., 1978; Rosenzweig and Bennett, 1996). This experimental paradigm is based on rearing the animals (typically rodents) in conditions in which the laboratory standards are enhanced by manipulating factors that mimic the ones considered reserve builders in humans, namely, the cognitive factor (objects multifarious in nature, shape, size, and color, frequently rearranged and replaced to expose the animals to the novelty); the social factor (increased number of individuals in the same cage in comparison to the laboratory standards); and the physical factor (cages bigger than the standard; shelves, ladders, and running wheels; supplementary diets; etc.) (Mandolesi et al., 2017; Sampedro-Piquero and Begega, 2017; Balietti and Conti, 2022). Animal models allow the investigation of the reserve in all its components, such as the brain, cognitive, and neural components. Furthermore, the use of animals makes available the direct investigation of a large number of structural, biological, and behavioral indices, also ensuring the possibility of high-level control of experimental variables (Petrosini et al., 2009; Gelfo et al., 2018).

The reserve mechanisms have been studied directly in the brain, and several studies have documented the association between different levels of reserve and brain structural/functional changes in several neurological conditions. In contrast, the relationship between reserve measures and the cerebellum has been described in a handful of studies and specifically investigated to a lesser extent (Mitoma et al., 2020; Bordignon et al., 2021). More focused evidence on the relationship between reserve measures and cerebellar structures is available in animal models (Gelfo and Petrosini, 2022a,b). This situation is on the whole very surprising since the plastic properties of the cerebellum are currently well-known (Luciani, 1893; Carulli et al., 2004; Cesa and Strata, 2005; Bosman and Konnerth, 2009; Cheron et al., 2016; Mitoma et al., 2021). Cerebellar involvement in cognitive and emotional functions in addition to motor functions has been increasingly ascertained (Schmahmann, 1997; Laricchiuta et al., 2015; Jang and Kim, 2019; De Zeeuw et al., 2021; Picerni et al., 2021, 2022; Petrosini et al., 2022; van Dun et al., 2022). Schmahmann (2019) reports that the cerebellum maintains behavior (in terms of cognition and emotions) around a homeostatic baseline by unconsciously using implicit learning and by adapting actions to context. The cerebellar posterior lobe plays a role in maintaining cognitive and emotional functions. Indeed, posterior lobe lesions produce cerebellar cognitive affective syndrome characterized by executive dysfunctions, visuospatial disorders, language difficulty, and affective dysregulation (Schmahmann, 2019). Distributed cerebro-cerebellar networks are involved in motor, cognitive, and emotional functions. In particular, the dorsal and ventral attentional networks, the frontoparietal network, the default mode network, and the salience network have been mapped into focal areas of the posterior lobe of the cerebellum. This cortico-cerebellar organization accounts for the functional connectivity between the cerebellum and associative brain areas that are involved in higher-level behaviors (Schmahmann, 2019).

A recent study investigated the association between cognitive reserve and several brain structures, including the cerebellum, in a cohort of young healthy subjects (Conti et al., 2021). A total of 77 healthy individuals with a median age of 27.5 years were recruited and underwent the Cognitive Reserve Index, a comprehensive questionnaire investigating several cognitive reserve factors, including education, occupation, and leisure activities. The authors found associations between the Cognitive Reserve Index and the gray matter volume of the left cerebellum. Moreover, they also found low functional connectivity of the right cerebellum with the executive control network (Conti et al., 2021).

In addition, a large number of animal studies have indicated that exposure to environmental enrichment is able to induce plastic rearrangements in the cerebellum of healthy rodents. Such plastic changes are associated with the potentiation of cognitive performance (Gelfo and Petrosini, 2022b). Most studies have investigated the effects of exposure to enhanced stimulation at an early age by reporting changes in cerebellar synaptogenesis (De Bartolo et al., 2015; Kim et al., 2019), neurotrophin and neurotransmitter expression (Naka et al., 2002; Angelucci et al., 2009; Vazquez-Sanroman et al., 2013), chromatin levels (Uphouse, 1978), and electrophysiological activity (Eshra et al., 2019). However, the provided frame is not fully clarified because of some conflicting results. A lack of effects following early exposure to environmental enrichment is reported in relation to cerebellar synaptogenesis (Nithianantharajah et al., 2004; Pascual and Bustamante, 2013), neurotrophin and neurotransmitter expression (Naka et al., 2002; Vazquez-Sanroman et al., 2013), and chromatin levels (Uphouse and Tedeschi, 1979). Scarce evidence is available in relation to the neuroprotective effects of environmental enrichment when the animals are exposed later in life. Nevertheless, some indications suggest a beneficial effect on spatial learning associated with changes in cerebellar volume and polypeptide levels (Horvath et al., 2015; Scholz et al., 2015).

In this view, it is possible to advance the specific and complex construct of the cerebellar reserve, which includes, on the one hand, the capacity of the cerebellar structure to efficiently compensate for the damage and, on the other hand, the neuroprotective effects of enhanced experiences on cerebellar structure (Mitoma et al., 2020, 2022; Bordignon et al., 2021; Gelfo and Petrosini, 2022a,b). As a consequence, the present review aims to analyze the available literature on the neuroprotective effects of enriched experiences in humans and animals on the occurrence of neural damage that involves cerebellar functionality. To address this issue, we considered only the studies that specifically investigated the association between the current concept of brain/cognitive/neural reserve and the cerebellum.

## 2. Neuroprotective effects of experience on the cerebellum in humans

Since the seminal work of Schmahmann, “The Cerebellum and Cognition” (1997), it has become clear that the cerebellum exerts a concomitant role in motor and cognitive functions (Koziol et al., 2014). While in animal models, the cerebellar plastic response to complex stimulations is widely documented (Cutuli et al., 2011; Gelfo and Petrosini, 2022a,b), in humans, it is less clear whether the cerebellum is sensitive to environmental stimuli to the point of changing its structures and/or function. Neuroimaging studies suggest that compensatory cerebellar reorganization might be present in neurodegenerative diseases, such as Alzheimer's disease (AD). Although several studies have documented cerebellar involvement in cognitive and emotional processes (Toniolo et al., 2018, 2020; Olivito et al., 2020; Petrosini et al., 2022), few studies have directly investigated the role of the cerebellum in the framework of brain/cognitive/neural reserve in clinical populations. Serra et al. (2017a) found a significantly decreased brain functional connectivity in a large network involving fronto-temporo-cerebellar nodes by comparing a group of patients with amnestic-mild cognitive impairment (a-MCI) and high educational level (a proxy measure of cognitive reserve) against a-MCI patients with low CR. In particular, significant differences in the correlations between pairs of nodes in a-MCI patients with high cognitive reserve have been found mainly between bilateral BA10 and bilateral cerebellar lobule 9, bilateral BA10 and cerebellar vermis, bilateral BA43 and cerebellar vermis, and, finally, right BA37 and right cerebellar lobule 10. In the same study, abnormalities in the local circuit properties in several nodes of the cerebellum were found in a-MCI and in AD patients with high cognitive reserve. Specifically, the betweenness centrality and the nodal degree (both measures of the efficiency of the cerebral–cerebellar networks; the betweenness centrality is calculated as the ratio of all the shortest paths passing through a given node; the nodal degree is the number of connections for each node) have been found to be reduced in bilateral lobule 10 and the left Crus II in a-MCI patients with high cognitive reserve compared with a-MCI patients with low cognitive reserve. In addition, healthy elderly controls with high cognitive reserve compared with those with low cognitive reserve showed reduced nodal degree in Crus I and Crus II and in lobules 8 and 9. Finally, AD patients with high cognitive reserve showed reduced nodal efficiency in the vermis (Serra et al., 2017a). The authors concluded that the reduced connectivity in this large network involving the cerebellum accounted for the cognitive disorders observed in the patients according to the cognitive reserve framework. Indeed, the cognitive reserve model postulates that individuals with high cognitive reserve levels need to accumulate more neuropathology than those with lower cognitive reserve levels to show the same rate of cognitive dysfunction (Stern and Barulli, 2019).

In a recent study on the relationship between structural changes in the brain and the cerebellar structure (Serra et al., 2017b), the cerebellum was found to be related to cognitive reserve (computed through a latent variable derived from memory and general cognition scores). In this study, direct correlations between cognitive reserve measures and gray matter volumes of the cerebellum, mainly in Crus I and lobules 4 and 7, were found in both a-MCI patients and healthy controls (Serra et al., 2017b).

A recent FDG-PET study (Canosa et al., 2021) on patients with amyotrophic lateral sclerosis (ALS) found an inverse correlation between the metabolism of the medial frontal lobe and the cerebellum only in the patient group and not in the control group. The authors hypothesized that this finding might be due to the recruitment of cerebellar reserve networks (Canosa et al., 2021).

Even in multiple sclerosis (MS), it has been described that there is a relationship between the cerebellum and cognitive reserve (Rocca et al., 2019). Namely, in patients with MS, an association was found between gray matter atrophy of the left cerebellum and memory and cognitive reserve measures. The authors concluded that in MS, cognitive reserve plays a protective role in preserving cognition, moderating the effect of structural damage on memory performance without affecting the progression of the disease (Rocca et al., 2019).

Details on the studies cited in this section are provided in Table 1.

**Table 1:** Studies on the neuroprotective effects of the exposure to enhanced stimulations in human neuropathological conditions involving cerebellar functionality.

**References**	**Subjects**	**Clinical diagnosis**	**Cognitive reserve measures**	**Effects on cerebellar structures**
Serra et al. (2017a)	*N* = 154; M/F = 63/91; Mean (SD) age = 70.1 (7.5); mean (SD) education = 10.8 (2.0)	AD = 68; a-MCI = 61; HS = 25	*z*-scores derived by years of formal education	Decreased functional resting-state connectivity in several cerebellar nodes
Serra et al. (2017b)	*N* = 82; M/F = 39/43; Mean (SD) age = 70.1 (7.0); mean (SD) education = 11.5 (4.0)	a-MCI = 34; HS = 48	Latent variables extracted by six episodic memory scores and mini mental state exam score	Direct correlations with cerebellar gray matter volumes
Canosa et al. (2021)	*N* = 111; M/F = 63/48; Median age = 63.5; median of education = 11	ALS = 111	Years of formal education	Negative correlation in metabolism between cerebellum and frontal regions
Rocca et al. (2019)	*N* = 74; Median age MS = 45.0; Median age HS = 42.3; Median years of formal education MS = 13; Median years of formal education HS = 16	MS = 54; HS = 20	Cognitive reserve Index questionnaire	Decreased gray matter volume of cerebellum

AD, Alzheimer's disease; a-MCI, amnestic mild cognitive impairment; ALS, amyotrophic lateral sclerosis; HS, healthy subjects; MS, multiple sclerosis; N, number; SD, standard deviation. See text for further details.

## 3. Neuroprotective effects of experience on the cerebellum in animal models

By analyzing animal studies focused on the neuroprotective effects of exposure to enhanced environmental stimulations before the occurrence of brain damage that affects cerebellar functionality and cognition, it is possible to detect that this issue is still scarcely addressed. Some evidence is provided about the neuroprotective effects of environmental enrichment in models of the Rett syndrome and cerebellar trauma. However, the reported findings are not completely consistent.

Rett syndrome is characterized by the progressive loss of motor and cognitive abilities, previously acquired in the early postnatal phases. It is caused by mutations in the X-linked methyl CpG-binding protein 2 (Mecp2) gene and is predominant in females. Such a disease is mimicked in Mecp2 mutant mouse models showing most impairments that characterize Rett syndrome patients, namely, deficits in motor, cognitive, social, and emotional competencies (Harris, 2021). By using one of these models—male Mecp2tm1Jae mice—Lonetti et al. (2010) reported that early exposure to environmental enrichment from 10 days of age for 50 days prevented the impairment in motor learning shown by standard-reared male homozygous Mecp2 mutant mice. This functional neuroprotective effect was accompanied by the increased density of cerebellar inhibitory synapses shown by the enriched mutant mice compared with the standard-reared mice. Moreover, in female heterozygous Mecp2 mutants, the same protocol of environmental enrichment prevented deficits in spatial learning and anxiety-related behaviors. Conversely, Nag et al. (2009) reported that the exposure to environmental enrichment of male Mecp2 mutant mice from 21 to 44 days of age did not improve the performance in contextual or cued fear conditioning, although it ameliorated the locomotor deficits. In association, it did not affect cerebellar volume.

A series of studies have been dedicated to the analysis of the neuroprotective effects of long-term exposure to environmental enrichment in a rat model of hemicerebellectomy, realized by surgically ablating half of the vermis and one entire cerebellar hemisphere of rats (Burello et al., 2012). Typically, the lesion induces postural and locomotor asymmetries that are almost completely compensated in ~3 weeks, accompanied by persistent impairment in complex motor behavior, spatial learning, and memory performance (Foti et al., 2011). Spontaneous cerebellar compensation is associated with plastic rearrangements in Purkinje cell spine size and density (Gelfo et al., 2016) and in the expression of nerve growth factor (NGF) and brain-derived neurotrophic factor (BDNF) in the spared hemicerebellum (Gelfo et al., 2011). By using this model, Foti et al. (2011) showed that the exposure to environmental enrichment from weaning onward induced an anticipation of locomotor and postural compensation of ~1 week in animals submitted to hemicerebellectomy on the 75th postnatal day. In addition, impairments in complex motor abilities and spatial learning and memory were almost completely prevented (Foti et al., 2011; Gelfo et al., 2016). Regarding morphological findings, Gelfo et al. (2016) reported that enriched rats maintained the amelioration of Purkinje cell dendritic spine size and density induced by environmental enrichment, as also shown by the enriched unlesioned animals, without displaying the additional increase induced by the lesion. Regarding the expression of neurotrophins, Gelfo et al. (2011) similarly showed that the expression of BDNF was not additionally increased in the spared hemicerebellum of the enriched lesioned animals with respect to the standard-reared lesioned animals. Such a finding indicates that structural and molecular upheavals are not necessary for enriched brains. The exposure to environmental enrichment ideally shapes neural connectivity, making further rearrangements unnecessary in cases of lesions. However, hemicerebellar NGF levels were additionally increased by the exposure to environmental enrichment and by the lesion.

Finally, it is appropriate to add the findings of some studies investigating the specific neuroprotective effect of a single component of environmental enrichment, such as exercise, in a rat model of chronic cerebral hypoperfusion. Notably, chronic cerebral hypoperfusion, thought to be the main cause of vascular dementia, is caused by brain thromboembolic events and induces progressive cognitive decline (Lee et al., 2021). By using a model of bilateral common carotid artery occlusion executed in adult rats (12 weeks of age), Lee et al. (2018) reported that the exposure of the animals to treadmill exercise from 4 weeks of age for 8 weeks reduced the deficits in spatial navigation due to subsequent chronic cerebral hypoperfusion. Such a cognitive effect was accompanied by reduced levels of reactive astrocytes and microglial activation in the cerebellum, reduced loss of Purkinje cells, and apoptotic cell death in the cerebellum. In a subsequent study (Lee et al., 2021) in the same model, it was demonstrated that preventive exposure to treadmill exercise from 4 weeks of age for 8 weeks induced a reduction in spatial working memory impairment associated with enhanced mitochondrial calcium retention capacity.

Details on the studies cited in this section are provided in Table 2.

**Table 2:** Studies on the neuroprotective effects of the exposure to enhanced stimulations in neuropathological animal models involving cerebellar functionality.

**References**	**Species and model of disease (age at the start of environmental enrichment)**	**Kind of enhanced stimulation (duration)**	**Enhanced stimulation effects on cognitive abilities and cerebellar structure (age at the evaluation)**
Nag et al. (2009)	Male Mecp2 mutant mice; model of Rett syndrome (21 days)	Environmental enrichment – with running wheels; – without social manipulation (23 days)	Unchanged contextual or cued fear conditioning (29–43 days); unchanged volume (44 days)
Lonetti et al. (2010)	Male homozygous and female heterozygous Mecp2 mutant mice; model of Rett syndrome (10 days)	Environmental enrichment – with running wheels (50 days)	– In males: prevention of deficits in motor learning (30–60 days); increased inhibitory synaptic density (52 days) – In females: prevention of deficits in spatial learning and amelioration of anxiety-related behaviors (60–75 days)
Foti et al. (2011)	Male Wistar rats; model of cerebellar trauma (hemicerebellectomy at 75th postnatal day) (21 days)	Environmental enrichment – with running wheels (about 3,5 months)	Prevention of deficits in spatial learning and memory (about 3–4 months)
Gelfo et al. (2011)	Male Wistar rats; model of cerebellar trauma (hemicerebellectomy at 75th postnatal day) (21 days)	Environmental enrichment – with running wheels (about 4 months)	Increased NGF expression; unchanged BDNF expression (5 months)
Gelfo et al. (2016)	Male Wistar rats; model of cerebellar trauma (hemicerebellectomy at 75th postnatal day) (21 days)	Environmental enrichment – with running wheels (about 4 months)	Increased spatial learning and memory performance (about 4 months); maintenance of Purkinje cell dendritic spine density and size (about 4.5 months)
Lee et al. (2018)	Male Wistar rats; model of chronic cerebral hypoperfusion (bilateral common carotid arteries occlusion at 12 weeks) (4 weeks)	Treadmill exercise protocol – 30 min/day; 2 m/min for the first 5 min, 3 m/min for the following 5 min and 5 m/min for the last 20 min, at 0 degrees of inclination (8 weeks)	Reduced spatial navigation impairment; decreased levels of reactive astrocytes and microglial activation; reduced apoptotic cell death; attenuated loss of Purkinje cells (about 3 months)
Lee et al. (2021)	Male Wistar rats; model of chronic cerebral hypoperfusion (bilateral common carotid arteries occlusion at 12 weeks) (4 weeks)	Treadmill exercise protocol – 30 min/day; 2 m/min for the first 5 min, 3 m/min for the following 5 min and 5 m/min for the last 20 min, at 0 degrees of inclination (8 weeks)	Reduced spatial working memory impairment; decreased loss of Purkinje cells; enhanced mitochondrial calcium retention capacity (about 4 months)

NGF, neurotrophic growth factor; BDNF, brain-derived neurotrophic factor.

## 4. Discussion

Until now, the elective locus of studies on reserve mechanisms has been the whole brain. Most studies have been driven by the evidence that different levels of reserve are associated with brain structural/functional changes in several neurological conditions. However, the specific relationship between reserve measures and individual brain areas has been scarcely described. In particular, only sporadic studies have addressed the link between reserve mechanisms and the cerebellum. This is very surprising, given the well-known plastic properties of the cerebellar networks and their involvement in high-level cognitive and emotional functioning. In recent years, some authors have addressed the specific construct of the cerebellar reserve. This construct, in accordance with the recent literature, encompasses both the capacity of the cerebellar structure to successfully compensate for the damage and the neuroprotective effects of lifelong enhanced experiences on the cerebellar structure. The present review falls within this framework and summarizes the available evidence on the neuroprotective effects of enriched experiences in humans and animals in pathological conditions involving cerebellar functionality. To this end, we included studies that documented the association between the current concept of the brain/cognitive/neural reserve and the cerebellum.

Such a literature review allowed us to reveal in both humans and animals the existence of reserve mechanisms specifically involving the structure, biology, and functionality of the cerebellum. However, these studies are still scarce and do not exhaustively deepen our understanding. Moreover, the evidence provided is not completely clear since the findings reported are sometimes conflicting. According to the key role of the cerebellum in the regulation of high-level cognitive and affective processes, the reported suggestions emphasized the importance of planning specific investigations disentangling the peculiar involvement of the cerebellum in reserve mechanisms. Such an issue has to be addressed in terms of both the dynamics and extension of the reserve development process. It is also important to promote the study of cerebellar reserve mechanisms in relation to the actions of different and specific reserve builders to design more personalized and tuned prevention and rehabilitation interventions in both healthy and pathological populations.

## Author contributions

All authors contributed to the design and conceptualization, literature search, and writing of the review and approved the final submitted version.
